# The Eye of the Chiropterologist: Phenotypic Versus Genotypic Identification of Bats

**DOI:** 10.1002/ece3.71561

**Published:** 2025-06-17

**Authors:** F. D. Dami, T. E. Adeyanju, A. A. Chaskda, I. M. Okpanachi, A. T. Adeyanju, S. M. Ezekiel, T. Gwom, I. A. Iniunam, A. Hitch, D. D. Pam, P. Luka, S. C. Weaver, S. Paessler, R. W. Cross, N. Shehu

**Affiliations:** ^1^ Department of Zoology University of Jos Jos Nigeria; ^2^ AP Leventis Ornithological Research Institute (APLORI) University of Jos Biological Conservatory Jos East LGA Plateau State Nigeria; ^3^ University of Ibadan Ibadan Nigeria; ^4^ University of Texas Medical Branch Galveston Texas USA; ^5^ National Veterinary Research Institute (NVRI) Vom Nigeria; ^6^ Jos University Teaching Hospital Jos Nigeria

**Keywords:** Chiroptera, genetic diversity, genotypic, identification, mitochondria, phenotypic

## Abstract

Bats are a diverse and ecologically important group of mammals that play critical roles in ecosystems. Accurate identification is necessary to comprehend bat species' ecology and behavior to further the conservation of bats. Both phenotypic and genotypic methods have been used for bat identification, but their relative effectiveness remains unclear in the Afrotropics. This study compared the advantages and limitations of phenotypic and genotypic identification of bats to improve and ensure effective bat species identification. Bats were captured using mist nets within protected and unprotected areas in different vegetation zones in Nigeria. Morphological identification of all captured bats was done using the guide, Mammals of Africa. Genotypic identification was done by extracting genomic DNA and Sanger sequencing of the generated mtDNA PCR amplicons. We then compared the sensitivity, specificity, positive predictive value (PPV) and negative predictive value (NPV) of the phenotypic to the genotypic outcomes of our identification. We trapped 91 bats, and the phenotypic identification of 90 individual species showed sensitivity ranges between 68% and 100%, except for *Glauconycteris* spp., whose sensitivity was low (14%). The specificity was generally good for all species > 96%. Phenotypic identification is accurate and reliable for most trapped bat species (*Epomorphorus gambianus*, *Scotophilus* spp., *Micropteropus pusillus* , *Rhinolophus* spp., *Roussettus aegyptiacus*, and *Chaerephon* spp.). However, phenotypic identification reveals its limitations in some bat species such as *Banana pipistrellus* and *Glauconycteris* spp., which had more variable results from their genetic characterization. *Epomorphorus gambianus* and *Micropteropus pusillus* had no distinct genetic differentiation in their mtDNA. This highlights the importance of using multiple methods for bat identification to ensure the most accurate results.

## Graphical abstract

This study compared the accuracy of phenotypic and genotypic identification methods for bats in Nigeria, revealing that while phenotypic identification is generally reliable for most species, it has limitations for certain species like *Glauconycteris* spp. The findings emphasize the importance of using both methods to ensure accurate bat species identification.

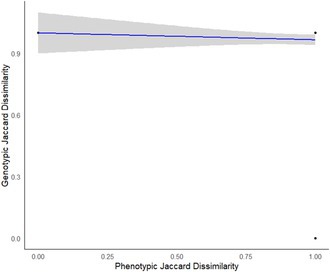

## 1 Introduction

Bats are an important group of mammals that play crucial ecological roles including insect control, pollination, seed dispersal, and nutrient cycling in various ecosystems of the world (Ramírez‐Fráncel et al. 2022). There are currently 1482 bat species in the world (Simmons and Cirranello 2024) with 404 of these known from Africa (Africa Chiroptera Report 2024). Bats are incredibly diverse morphologically and physiologically, resulting in varied ecological adaptations (Monadjem et al. 2018).

Identifying bats can be challenging due to their small size and very similar morphological resemblance among species conspecifics (Frick et al. 2020; Russo and Voigt 2016). Therefore, morphological, acoustic, and genotypic characterizations are commonly used for bat identification (Dietz and von Helversen 2004; Grenié et al. 2023; Hassanin 2014; Zamora‐Gutierrez et al. 2016).

The use of morphological, acoustic, and mitochondrial data in concert has been significantly more efficient than either of these used in isolation of the other (Hassanin 2014; Hassanin et al. 2015; Foley et al. 2017; Tanshi et al. 2022). The cost of identification for bats can be astronomical based on post‐capture costs; vouchers need to be taken to the laboratory and museums for cross‐matching when dependent solely on morphological parameters. When molecular techniques are followed, real‐time or next‐generation PCR machines are relatively accessible; however, sequencing large data sets could be very expensive. Data sharing among researchers is another challenge; not all sequences are available in the NCBI database for cross‐matching. Acoustic detection tools are also dependent on manually identifying hand‐released bats.

Re‐examining previous species distributions and checklists of Nigeria has resulted in splits of past groupings, such as the *Rhinolophus commersoni* complex (Hassanin et al. 2015; Foley et al. 2017) into Macronycteris *gigas* and *M. vitatus*, formerly unknown bat species in Nigeria (Happold 1987). Molecular, morphological, and acoustic tools have improved these revisits on taxonomy. Similarly, the *Scotonycteris* complex was addressed by Hassanin et al. (2015); therefore, a full species *S. occidentalis* has been upgraded from a subspecies based on phylogeography and reported by Tanshi et al. (2022), Mulvaney et al. (2023), Hassanin (2014), Nesi et al. (2011), Goodman et al. (2009), Arnaout et al. (2022).

Deploying microsatellite and mitochondrial DNA analysis tools can provide definitive species identification and help reconstruct phylogenetic relationships among little known species (Chauhan et al. 2022; Mota et al. 2018). However, the technique is expensive and seldom used in developing countries for wildlife research.

Furthermore, the accuracy of bat identification using any of these methods can be compromised by the use of outdated taxonomic names and inconsistent species concepts (Tsang et al. 2016). The capacity to define, measure, and monitor ecological communities over time and place is essential for effective conservation (Magurran et al. 2010; Zamora‐Gutierrez et al. 2016). However, the taxonomy of bats has undergone significant revisions in recent years, resulting in changes in species names, distributions, and inferred phylogenetic relationships (Tsang et al. 2016). The latter can make it difficult to compare and synthesize data from different studies, particularly when the taxonomy used in a study is outdated or genetic sequences to compare upcoming research are unavailable (Miles and Dunham 1993). In addition, the lack of standardization in data sharing and the reluctance of researchers to share raw data even when available can hinder the reproducibility of studies and limit the use of existing data for meta‐analyses and global assessments of bat populations (Garrett‐Ruffin et al. 2021).

Bat diversity in the African biosphere has recently attracted attention (Herkt et al. 2016). There have been arguments and questions (Adeyanju et al. 2017) about the sub‐division of the Order Chiroptera after recent comparative and molecular studies (Nikaido et al. 2020). Unfortunately, little is known about the variety, distribution, and ecological needs of bats, making them one of the least understood mammalian groups in Nigeria (Adeyanju et al. 2017). Valid identification of bats to species is invaluable in tracking bat species that are known to be carriers of emerging zoonotic infectious diseases (Simmons and Cirranello 2024). Hence, this study sought to test the consistency, reliability, and integrity of phenotypic and genotypic identification of some bat species in Nigeria, and assess its genetic diversity.

## 2 Materials and Methods

### 2.1 Study Area

**FIGURE 1:**
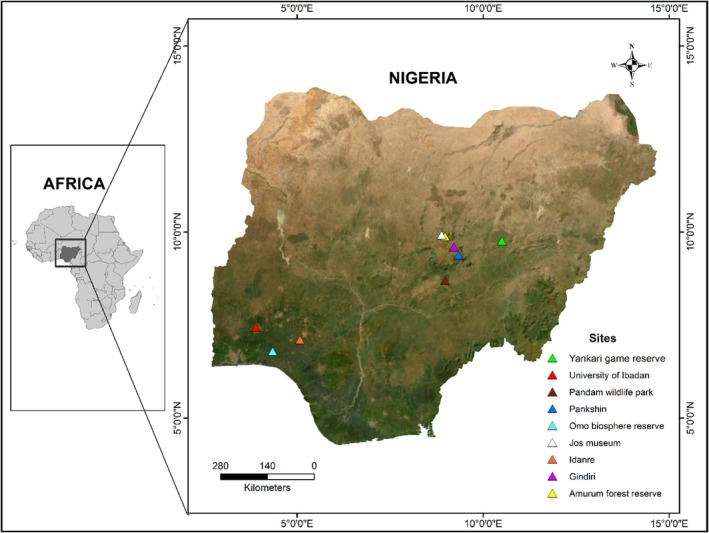
Map of Nigeria with the sampled location.

Bats were sampled across different sites in Nigeria. Sites sampled include the Amurum Forest Reserve, Pandam Wildlife Park, Gindiri, Jos Museum, Pankshin (guinea savanna), the Omo‐Biosphere Reserve (Forest), Idanre and the University of Ibadan (derived‐guinea savanna vegetation), and Yankari Game Reserve, which is within the Sudan savanna (See Figure 1).

### 2.2 Bat Trapping Technique

We selected 15 points where we set up 15 mist nets at the various sampling sites. We ensured that nets were set at least 20 m apart from each other, along forest tracks, across water pools, and around identified bat roosts. Nets were set up and opened after sunset (1800–2300 h) and before sunrise from (0400–0630 h). To reduce net destruction and escape time, we carried out routine net checks every 5 min. Bats were extracted and placed in an ecotone bag before being processed for sample collection. Morphological characteristics, body size, forearm length, sex, age (juvenile and adult), and reproductive status (breeding or non‐breeding) were noted (Adeyanju et al. 2017). Each bat's morphological identification was done using the guide, Mammals of Africa: Volume IV: Hedgehogs, Shrews, and Bats (Happold and Happold 2013).

### 2.3 Specimen Collection

We manually restrained the bats on a flat surface using thick hand gloves placed on the head and limbs to avoid injury from the defensive bites and associated claws of the bats. We opened the wings of trapped bats, and with the help of a sterile scalpel and scissors, a portion (about ~8 mm) of the wing was cut and gently placed in a sterile, airtight cryovial tube and stored at −20°C in the field and subsequently at −80°C before further analysis. All the bats were released back into the environment immediately after specimen collection was completed.

### 2.4 DNA Extraction, PCR Amplification, and Sequencing of COI Mitochondrial Genes

Following the Zymo Quick‐DNA Miniprep Extraction Kit protocol, we extracted total genomic DNA from the tissue sample. The eluted DNA was stored at −20°C until used for PCR amplification. The mitochondrial COI gene was amplified using the following set of oligonucleotide primers: 5′‐CCTACTCRGCCATTTTACCTATG‐3′ and 5′‐ACTTCTGGGTGTCCAAAGAATCA‐3′ (Mifsud and Vella 2019).

The Polymerase Chain Reaction (PCR) was carried out in a 25 μL reaction volume containing a 5 μL of 5X FIREPOL Master Mix containing 12.5 mM Mg^2+^, 1 mM of each dNTP, and 1 U FIREPOL DNA polymerase (Solis BioDyne, Estonia), and 0.5 μM of each primer. PCRs were carried out on a MiniAmp Thermal Cycler (Applied Biosystems) using the following temperature profile: 95°C for 5 min; followed by 35 cycles of 95°C for 45 s, 50°C for 30 s, 72°C for 1 min and final extension at 72°C for 15 min. From each PCR reaction, 2 μL of the PCR product was visualized on a 1.5% agarose gel stained with Gelred Nucleic Acid Gel Stain (Biotium) for an expected band size of 702 bp together with a 100 bp DNA ladder (NIPPON Genetics). PCR products were purified using Zymo DNA Clean and Concentrator (DCC‐25) and then sequenced in a single direction (amplification forward primer) using an automated DNA sequencer (Applied Biosystems 3500).

### 2.5 Mitochondrial DNA Sequence Analyses

Generated mtDNA sequences were carefully checked for ambiguous peaks in the chromatogram and trimmed to eliminate primer sequences using FinchTV (V 1.5. Geospiza Inc. n.d.), a chromatogram viewer. To confirm the phenotypic identification of bat species, newly generated sequences were compared to highly similar sequences available on the National Center for Biotechnology Information (NCBI) nucleotide standard database (Blast) based on percentage and query cover.

Sequences were aligned using MUSCLE (Edgar 2004), and phylogenetic relationships were estimated using Maximum Likelihood (ML) analyses. The Hasegawa‐Kishino‐Yano (HKY) model, identified as the best‐fitting nucleotide substitution model based on the Akaike Information Criterion (AIC) using MEGA (Tamura et al. 2021), was applied for constructing the phylogenetic tree. The Maximum Likelihood phylogenetic tree was computed using Geneious software, and branch support values are reported as percentages.

### 2.6 Phenotypic and Genotypic Identification of Bat Species

The sensitivity, specificity, Positive Predicted Value (PPV), and Negative Predicted Value (NPV) of the morphometric method of bat identification were determined against the genotypic method (Fabricant 2024; Trevethan 2017). The genotypic method served as the reference standard that could accurately identify bat species (Patrick et al. 2017). Sensitivity refers to the percentage of true positives that are correctly identified by the test, while specificity is the percentage of true negatives that are correctly identified by the test (Trevethan 2017). The PPV is the percentage of positive test results that are truly positive, and NPV represents the percentage of negative test results that were truly negative. The frequency (*n*) distribution also suggests that certain species are either more abundant or easier to identify accurately.

### 2.7 Statistical Comparison of Phenotypic and Genotypic Identifications

To assess the congruence between phenotypic and genotypic methods of species identification, we performed a Mantel test (Mantel 1967) using the vegan package (v2.6‐4) in R (v4.3.1). This test evaluates the correlation between two dissimilarity matrices: one derived from morphological trait‐based identifications and the other from mitochondrial DNA (mtDNA) barcoding. For both methods, we constructed binary (presence/absence) matrices with individuals as rows and species as columns. Jaccard dissimilarity indices, appropriate for presence/absence data, were calculated between all pairs of individuals within each matrix to quantify compositional dissimilarity. The Mantel test was then used to assess the relationship between these matrices using Pearson's correlation coefficient, with significance determined through 9999 permutations. A significant positive Mantel r value (ranging from −1 to 1) would indicate strong concordance between phenotypic and genotypic identifications.

## 3 Results

Ninety‐one individual bats were trapped and were first phenotypically identified, and subsequently genotypic identification was done (Table 1). Generated sequences have been deposited at GenBank with accession number OR545726–OR545795.

**TABLE 1:** Genotypic Identification of bat species with their corresponding phenotypic identification.

S/no	Phenotypic	Genotypic	Family	Sub‐family	Frequency (*n*)
1	*Banana pipistrellus*	*Hipposideros ruber*	Vespertilionidae	Vespertilioninae	1
2	*Banana pipistrellus*	*Neoromicia* spp.	Vespertilionidae	Vespertilioninae	1
3	*Banana pipistrellus*	*Neoromicia somalicus*	Vespertilionidae	Vespertilioninae	6
4	*Banana pipistrellus*	*Pipistrellus* spp.	Vespertilionidae	Vespertilioninae	4
**5**	***Epomophorus gambianus***	***Epomophorus gambianus***	Pteropodidae	Epomophorinae	**18**
6	*Glauconycteris* spp.	*Pipistrellus* spp.	Vespertilionidae	Vespertilioninae	3
7	*Glauconycteris* spp.	*Miniopterus* spp.	Vespertilionidae	Vespertilioninae	1
8	*Hipposideros jonesi*	*Hipposideros pomona*	Hipposideridae	Hipposiderinae	4
9	*Hipposideros* spp.	*Hipposideros ruber*	Hipposideridae	Hipposiderinae	3
10	*Hipposideros jonesi*	*Megaloglossus woermanni*	Hipposideridae	Hipposiderinae	1
**11**	***Megaloglossus woermanni***	***Megaloglossus woermanni***	Pteropodidae	Pteropodinae	**3**
12	*Megaloglossus woermanni*	*Hipposideros pomona*	Pteropodidae	Pteropodinae	1
**13**	***Micropteropus pusillus***	***Micropteropus pusillus***	Pteropodidae	Epomophorinae	**11**
**14**	**Pipistrellus spp**.	**Pipistrellus spp**.	Vespertilionidae	Vespertilioninae	**2**
15	*Rhinolophus landeri*	*Rhinolophus* spp.	Rhinolophidae	Rhinolophinae	1
**16**	***Rousettus aegyptiacus***	***Rousettus aegyptiacus***	Pteropodidae	Rousettinae	**1**
**17**	**Scotophilus spp**.	**Scotophilus spp**.	Vespertilionidae	Vespertilioninae	**18**
18	*Tadarida* spp.	*Chaerephon pumilus*	Molossidae	Molossinae	6
19	*Tadarida* spp.	*Chaerephon plicatus*	Molossidae	Molossinae	1
20	*Tadarida* spp.	*Chaerephon* spp.	Molossidae	Molossinae	5

*Note:* NB: Species in bold are species that were identified to be the same both genotypically and phenotypically.

**FIGURE 2:**
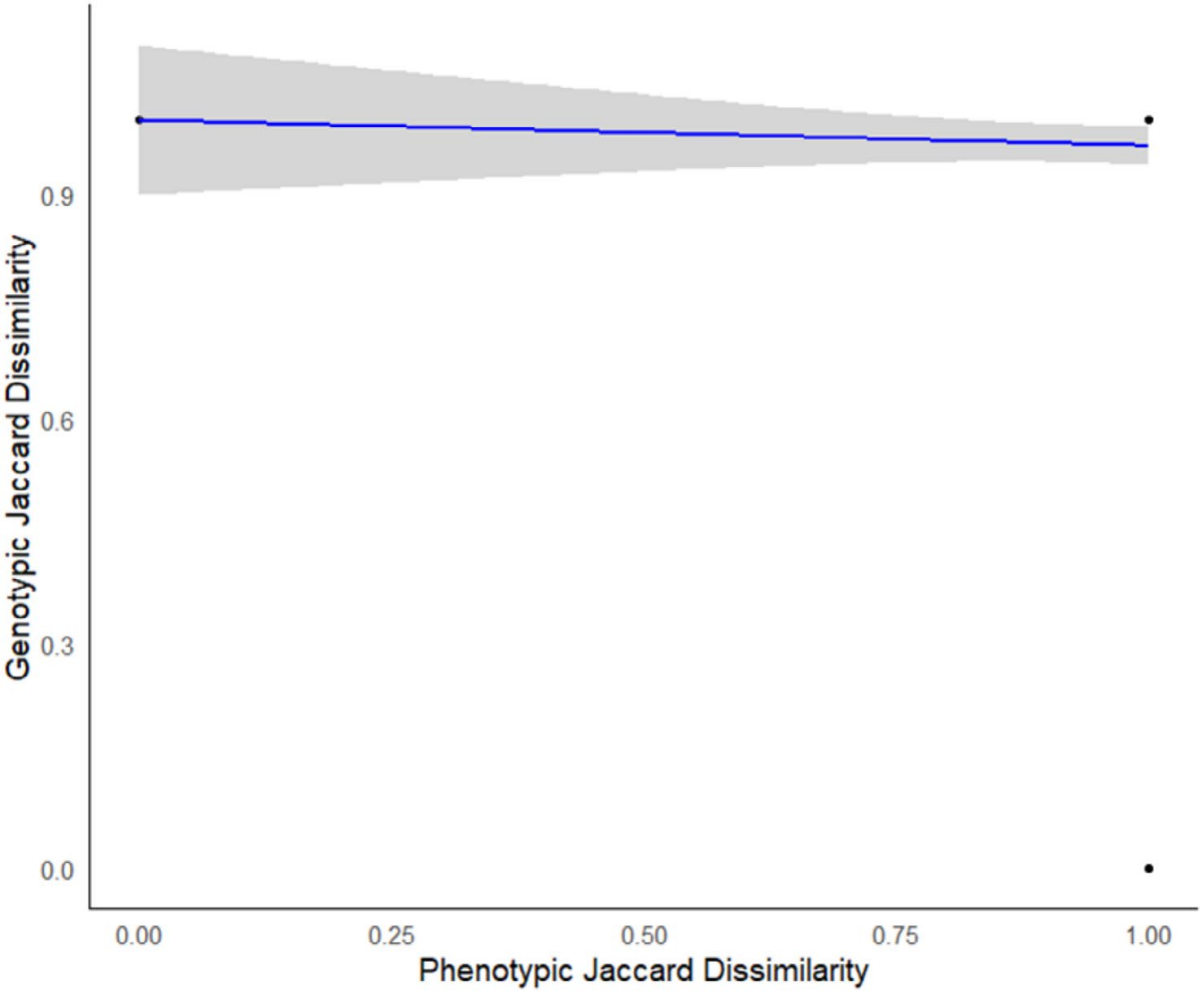
The relationship (blue line) between phenotypic and genotypic Jaccard dissimilarities with a 95% confidence (gray area). Mantel *r* = −0.047, *p* = 1.

Six out of the 20 incidents were identified to be the same species both genotypically and phenotypically (Table 1). These species are *Epomophorus gambianus, Megaloglossus woermanni, Micropteropus pusillus, Pipistrellus* spp., *Roussettus aegyptiacus, Scotophilus* spp. (Table 1). However, species that were identified as *Banana pipistrellus* and *Tadarida* spp. phenotypically were often different species genotypically (Table 1). The result indicated a weak negative and non‐significant correlation between the phenotypic and genotypic matrices (Mantel *r* = −0.047, *p* = 1.000), suggesting little to no concordance between the two methods of species identification (Figure 2).

## 4 Discussion

Our study shows that *Scotophilus* spp., *Neoromicia* spp., *Chaerephon* spp., *Epomophorus gambianus, Micropteropus pusillus, Rhinolophus* spp, and *Roussettus aegyptiacus* all had perfect sensitivity, specificity, PPV, and NPV scores of 100%, indicating that the phenotypic identification of these species was accurate and reliable. On the other hand, *Glauconycteris spp* had a low sensitivity and specificity of 14.29% and 96.81%. Similarly, *Pipistrellus spp* showed a relatively high specificity of 98.9%, but a lower sensitivity of 66.67%. *Hipposideros spp* and *Megaloglossus woermanni* have moderate to high sensitivity and specificity, as well as PPV and NPV values. Pipistrellus spp showed a relatively high specificity of 98.9%, but a lower sensitivity of 66.67%, indicating that the identification of this species was more reliable when identifying individuals that did not belong to the species. The lack of a significant correlation between phenotypic and genotypic identification methods highlights a substantial incongruence between morphological and molecular approaches in this study (Figure 2, Table 2). Our result suggests that individuals grouped similarly based on observable traits were not genetically similar, and vice versa. For example, species like *Glauconycteris* spp and *Pipistrellus* spp did not align using the two methods of identification, emphasizing the need for molecular validation in morphologically cryptic or taxonomically complex groups (Table 2). In the Afrotropics, where cryptic diversity is increasingly documented (Hughes et al. 2011), this finding reinforces concerns about the limitations of phenotypic identification alone, particularly in taxonomically complex groups such as bats.

**TABLE 2:** Sensitivity, specificity, positive predictive value (PPV), and negative predictive value (NPV) of the phenotypic identification of bat species.

Species	Sensitivity (%)	Specificity (%)	Positive predicting value (%)	Negative Predicting value (%)
*Scotophilus* spp	100.00	100.00	100.00	100.00
*Neoromicia* spp	100.00	100.00	100.00	100.00
*Epomophorus gambianus*	100.00	100.00	100.00	100.00
*Micropteropus pusillus*	100.00	100.00	100.00	100.00
*Rhinolophus* spp	100.00	100.00	100.00	100.00
*Rousettus aegyptiacus*	100.00	100.00	100.00	100.00
*Chaerephon* spp	100.00	100.00	100.00	100.00
*Pipistrellus* spp	66.67	98.90	85.71	96.77
*Glauconycteris* spp	14.29	96.81	14.29	96.81
*Hipposideros* spp	77.78	98.82	87.5	97.67
*Megaloglossus woermanni*	75.00	98.88	75.00	98.88

Morphological identification could be influenced or constrained by intraspecific variation, sexual dimorphism, age‐related changes, and observer subjectivity (Francis et al. 2010), thus impacting the accurate identification of species. Moreover, some bat species could exhibit convergent morphological traits due to shared ecological niches, which can obscure true genetic boundaries (Taylor et al. 2012). In contrast, DNA barcoding, particularly using mitochondrial markers like cytochrome *c* oxidase I (COI), has proven effective in resolving species boundaries, detecting cryptic species, and minimizing misidentifications in chiropteran taxa (Clare et al. 2007; Hebert et al. 2003).

Our study demonstrates a partial effectiveness of phenotypic identification for all bat species while also revealing discrepancies, particularly in *Glauconycteris* spp. and *Tadarida* spp., highlighting the limitations of relying solely on morphological traits, especially in species complexes. While some species were consistently identified by both methods, others exhibited mismatches, suggesting the limitation of using a single biomarker. Species morphological measurements overlap considerably, and many bat species share morphological attributes, which makes identification difficult to decipher. These findings highlight the necessity of integrating genetic analysis (multiple markers) with traditional non‐invasive morphological approaches to enhance species identification, which is essential for ecological research, disease surveillance, and conservation efforts.

**FIGURE 3:**
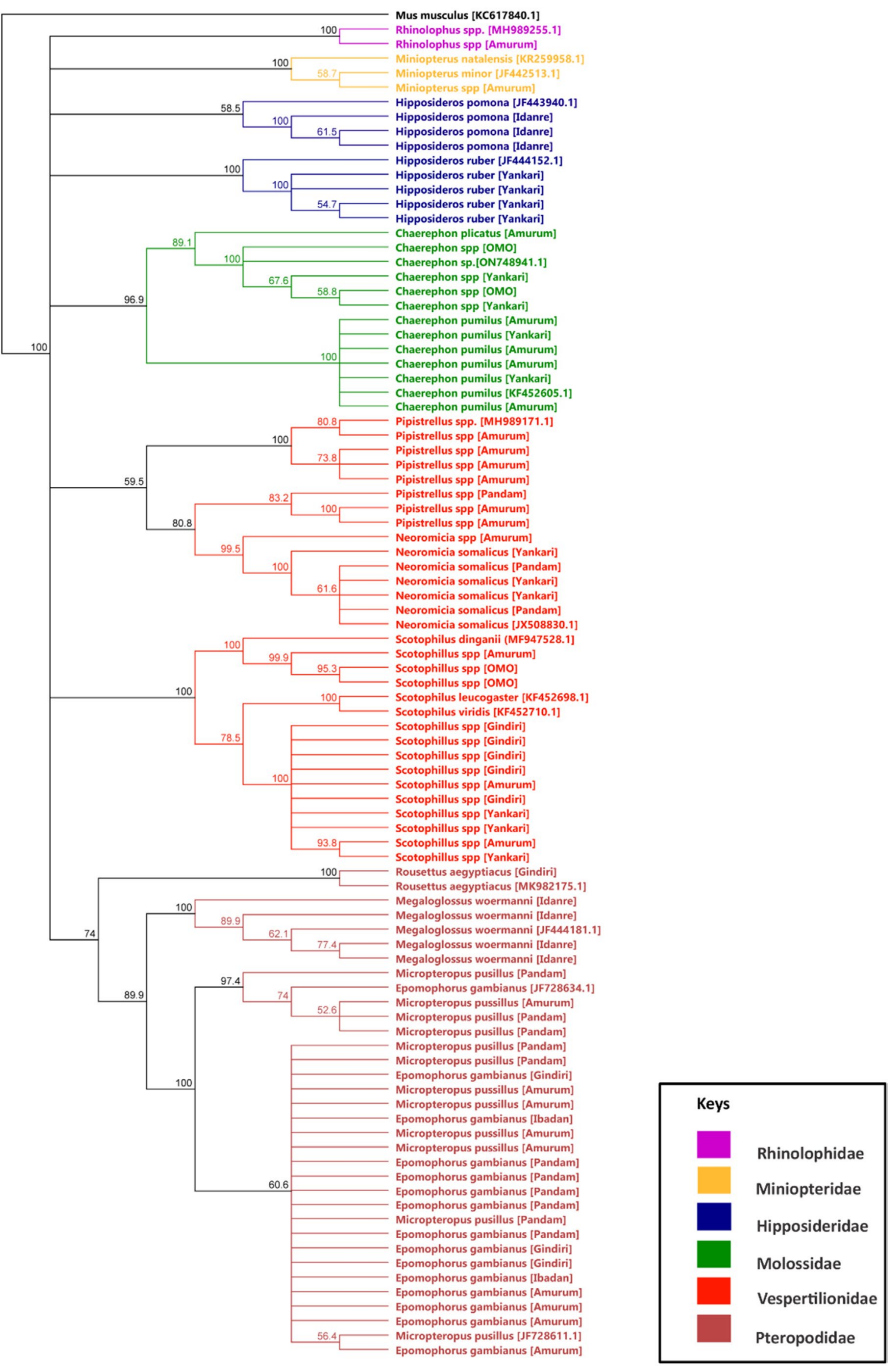
Maximum likelihood (ML) phylogenetic cladogram tree based on mtDNA generated sequence, reference sequence, and an outgroup sequence. Branch support values are shown if > 50%. The phylogenetic tree shows the evolutionary relatedness using the Hasegawa‐Kishino‐Yano (HKY) model.

We observed consistency between morphological identification and genetic identification for most species; however, more information on the fine‐scale species clarity, for example, the genus *Chaerephon* sampled at Amurum Forest Reserve, Omo‐Biosphere Reserve, and Yankari game reserve, showed similar morphological characteristics, hence classified in the same genus, but showed some level of cryptic diversity and/or close genetic similarity as displayed in Figure 3 (Russell et al. 2005; Stadelmann et al. 2004). In other groups, *Pipistrellus* spp., *Neoromicia* spp., *Hipposideros pomona* , *Hipposideros ruber* , *Chaerephon spp., Chaerephon pumilus*, *Chaerephon plicatus, Miniopterus* spp., *Scotophilus* spp., *Rousettus aegyptiacus* , and *Megaloglossus woermanni* formed distinct species clades (Figure 3). *Pipistrellus* spp., *Scotophilus* spp., *Neoromicia somalicus* showed genetic diversity based on the phylogenetic analysis. *Epomorphorus gambianus* (a large fruit bat) and *Micropteropus pusillus* (a small‐sized bat) revealed low genetic distances; a strange finding, however, corroborated by a study in the Central African Republic (Nesi et al. 2011). Phylogenetic analysis revealed evidence of genetic variation, with several polytomies and branches showing low bootstrap support, suggesting either rapid speciation events or unresolved lineages. These patterns may reflect ongoing evolutionary processes such as recent radiations or sympatric speciation driven by ecological or behavioral factors. For instance, bat groups exhibiting similar morphology but distinct lineages could be influenced by panmictic populations undergoing behavioral divergence. Alternatively, the observed polytomies may be attributed to insufficient sequence data or gaps in sampling, limiting the resolution of certain clades. To improve tree resolution and more clearly define lineages, future analyses could incorporate additional mitochondrial or nuclear markers and include reference sequences from closely related species within the same genera retrieved from public databases like NCBI.

Overall, the efficiency of DNA genotyping and barcoding in bat identification was validated compared to the sole use of traditional identification by morphological characteristics due to bat species overlapping features (Nesi et al. 2011).

Morphological identification is sufficient for species with high congruence (e.g., *Epomophorus gambianus, Scotophilus* spp.), but genetic methods are critical for resolving cryptic diversity (e.g., *Glauconycteris* spp.). Misidentification can lead to inaccurate assessments of species distributions or population trends. For example, *Banana pipistrellus* phenotypically identified individuals were genetically assigned to multiple genera (*Hipposideros, Neoromicia*), which could misdirect habitat protection efforts. Given these findings, integrating phenotypic and genotypic methods is essential for robust species identification. However, in regions with high biodiversity and limited taxonomic resolution, like the Afrotropics, genetic approaches may offer a more reliable baseline for species discovery and monitoring. This study underscores the need for expanded molecular reference libraries and highlights the limitations of relying solely on morphology or single markers in phylogenetic analysis in conservation, ecological, and biodiversity assessments (Teeling et al. 2018).

## 5 Conclusions

Clear‐cut taxonomical identification of bats can be achieved by complementary application of morphological and genotypic methods. Non‐invasive morphological identification has shown many limitations in identifying bats, underscoring the importance of genetic identification together with morphological identification of some genera of bat species. Morphological identification alone can be limited due to indistinguishable morphological features in juvenile bats and can lead to misidentifications. Our study has revealed that some bat species are consistently identified as the same species through both phenotypic and genotypic means. However, in some cases, mtDNA does not differentiate some species, for example, *Epomophorus gambianus* and *Micropteropus pusillus* (Nesi et al. 2011), and morphological identification in such cases can be relied upon in these instances. These findings are vital for bat ecologists since they validate their identification in the field. However, care needs to be taken and samples subjected to genetic identification for certain species such as *Pipistrellus* spp, *Neoromicia* spp., *Glauconycteris* spp., and *Chaerephon* spp, which were not consistent due to morphological similarity and/or taxonomic change. Hence, we advocate for a tiered approach: morphological identification for rapid surveys, supplemented by genetic barcoding based on multiple markers in cases of uncertainty or for taxonomically challenging groups.

## Author Contributions

**F. D. Dami:** conceptualization (lead), data curation (lead), formal analysis (lead), funding acquisition (equal), investigation (lead), methodology (lead), project administration (lead), resources (lead), software (lead), supervision (lead), validation (lead), visualization (lead), writing – original draft (lead), writing – review and editing (lead). **T. E. Adeyanju:** conceptualization (equal), data curation (equal), formal analysis (equal), investigation (equal), methodology (equal), project administration (equal), resources (equal), supervision (equal), validation (equal), visualization (equal), writing – original draft (equal), writing – review and editing (equal). **A. A. Chaskda:** conceptualization (equal), data curation (equal), formal analysis (equal), funding acquisition (equal), investigation (equal), methodology (equal), project administration (equal), resources (equal), software (equal), supervision (equal), validation (equal), visualization (equal), writing – original draft (equal), writing – review and editing (equal). **I. M. Okpanachi:** data curation (equal), formal analysis (equal), investigation (equal), methodology (equal), resources (equal), software (equal), visualization (equal), writing – original draft (equal), writing – review and editing (equal). **A. T. Adeyanju:** conceptualization (equal), data curation (equal), formal analysis (equal), investigation (equal), methodology (equal), resources (equal), supervision (equal), visualization (equal), writing – original draft (equal), writing – review and editing (equal). **S. M. Ezekiel:** data curation (equal), formal analysis (equal), investigation (equal), methodology (equal), resources (equal), writing – original draft (equal), writing – review and editing (equal). **T. Gwom:** conceptualization (equal), data curation (equal), formal analysis (equal), investigation (equal), methodology (equal), resources (equal), visualization (equal), writing – original draft (equal), writing – review and editing (equal). **I. A. Iniunam:** formal analysis (equal), visualization (equal), writing – original draft (equal), writing – review and editing (equal). **A. Hitch:** conceptualization (equal), funding acquisition (equal), investigation (equal), methodology (equal), project administration (equal), resources (equal), software (equal), supervision (equal), validation (equal), visualization (equal), writing – original draft (equal), writing – review and editing (equal). **D. D. Pam:** conceptualization (equal), funding acquisition (equal), project administration (equal), supervision (equal), writing – original draft (equal), writing – review and editing (equal). **P. Luka:** conceptualization (equal), funding acquisition (equal), investigation (equal), methodology (equal), project administration (equal), supervision (equal), validation (equal), visualization (equal), writing – original draft (equal), writing – review and editing (equal). **S. C. Weaver:** conceptualization (supporting), data curation (supporting), formal analysis (supporting), funding acquisition (supporting), investigation (supporting), methodology (supporting), project administration (supporting), resources (supporting), software (supporting), supervision (supporting), validation (supporting), visualization (supporting), writing – original draft (supporting), writing – review and editing (supporting). **S. Paessler:** conceptualization (equal), data curation (equal), formal analysis (equal), funding acquisition (equal), investigation (equal), methodology (equal), project administration (equal), resources (equal), supervision (equal), visualization (equal), writing – original draft (equal), writing – review and editing (equal). **R. W. Cross:** conceptualization (equal), data curation (equal), formal analysis (equal), funding acquisition (equal), investigation (equal), methodology (equal), project administration (equal), resources (equal), software (equal), supervision (equal), validation (equal), visualization (equal), writing – original draft (equal), writing – review and editing (equal). **N. Shehu:** conceptualization (equal), data curation (equal), formal analysis (equal), funding acquisition (equal), investigation (equal), methodology (equal), project administration (equal), resources (equal), software (equal), supervision (equal), validation (equal), visualization (equal), writing – original draft (equal), writing – review and editing (equal).

## Conflicts of Interest

The authors declare no conflicts of interest.

## Data Availability

The dataset for this paper is available on the NCBI database at https://www.ncbi.nlm.nih.gov/gene/—GenBank accession number OR545726—OR545795.
